# 3,3′-Dimethyl-1,1′-(propane-1,3-di­yl)diimidazol-1-ium bis­(1,2-dicyano­ethene-1,2-dithiol­ato-κ^2^ *S*,*S*′)nickelate(II)

**DOI:** 10.1107/S1600536811027693

**Published:** 2011-07-23

**Authors:** Shan-Shan Yu, Hai-Bao Duan, Xiao-Ming Ren

**Affiliations:** a Department of Chemistry, Nanjing Xiaozhuang College, Nanjing 210017, People’s Republic of China; b School of Biochemical and Environmental Engineering, Nanjing Xiaozhuang College, Nanjing 210017, People’s Republic of China; c College of Science, Nanjing University of Technology, Nanjing 210009, People’s Republic of China

## Abstract

In the title compound, (C_11_H_18_N_4_)[Ni(C_4_N_2_S_2_)_2_], the asymmetric contains one half-complex, with the cation placed on a twofold axis and the anion located on an inversion center. The Ni^II^ ion in the anion is coordinated by four S atoms of two maleonitrile­dithiol­ate ligands, and exhibits the expected square-planar coordination geometry.

## Related literature

For the design of functional materials, see: Robertson & Cronin (2002 ▶). For near-infrared dyes, conducting, magnetic and non-linear optical materials, see: Nishijo *et al.* (2000 ▶); Ni *et al.* (2005 ▶). For related structures, see: Ni *et al.* (2004 ▶); Ren *et al.* (2004 ▶, 2008 ▶); Duan *et al.* (2010 ▶); For the synthesis of the title compound, see: Davison & Holm (1967 ▶); Yao *et al.* (2008 ▶).
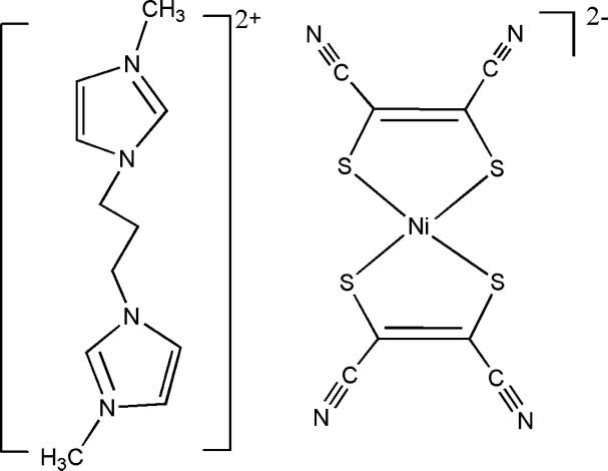


## Experimental

### 

#### Crystal data

(C_11_H_18_N_4_)[Ni(C_4_N_2_S_2_)_2_]
*M* *_r_* = 545.38
Monoclinic, 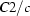

*a* = 19.3683 (15) Å
*b* = 7.3026 (6) Å
*c* = 17.5170 (14) Å
β = 104.167 (1)°
*V* = 2402.2 (3) Å^3^
*Z* = 4
Mo *K*α radiation
μ = 1.18 mm^−1^
*T* = 293 K
0.4 × 0.3 × 0.3 mm

#### Data collection

Bruker SMART CCD area-detector diffractometer
Absorption correction: multi-scan (*SADABS*; Bruker, 1999 ▶) *T*_min_ = 0.702, *T*_max_ = 0.741
7551 measured reflections
2938 independent reflections
2503 reflections with *I* > 2σ(*I*)
*R*_int_ = 0.067

#### Refinement

*R*[*F* ^2^ > 2σ(*F* ^2^)] = 0.037
*wR*(*F* ^2^) = 0.101
*S* = 1.06
2938 reflections
147 parameters
H-atom parameters constrained
Δρ_max_ = 0.31 e Å^−3^
Δρ_min_ = −0.68 e Å^−3^

### 

Data collection: *SMART* (Bruker, 1999 ▶); cell refinement: *SAINT* (Bruker, 1999 ▶); data reduction: *SAINT*; program(s) used to solve structure: *SHELXS97* (Sheldrick, 2008 ▶); program(s) used to refine structure: *SHELXL97* (Sheldrick, 2008 ▶); molecular graphics: *SHELXTL* (Sheldrick, 2008 ▶); software used to prepare material for publication: *SHELXTL*.

## Supplementary Material

Crystal structure: contains datablock(s) global, I. DOI: 10.1107/S1600536811027693/bh2362sup1.cif

Structure factors: contains datablock(s) I. DOI: 10.1107/S1600536811027693/bh2362Isup2.hkl

Additional supplementary materials:  crystallographic information; 3D view; checkCIF report

## Figures and Tables

**Fig. 1.**
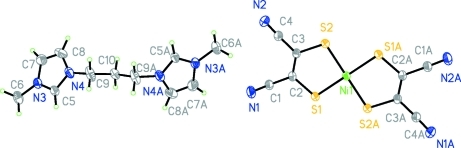
The molecular structure of the title compound, showing displacement ellipsoids at the 30% probability level.

## supplementary crystallographic information

### Comment

Supramolecular chemistry and molecular crystal engineering, which is the planning and utilization of crystal-oriented syntheses for the bottom-up construction of functional molecular solids from molecules and ions, are powerful tools for the assembly of designed functional materials (Robertson & Cronin, 2002). The use of bis-1,2-dithiolene complexes of transition metals as building units in the construction of such molecule based materials has received extensive attention due to their potential applications in the areas of near-infrared (near-IR) dyes, conducting, magnetic and nonlinear optical materials (Nishijo *et al.*, 2000; Ni *et al.*, 2005). Herein we report the crystal structure of the title compound (Fig. 1), which belongs to this class of materials.

The compound crystallizes in monoclinic system, with one half of [Ni(*mnt*)_2_]^2-^ dianion (*mnt* = maleonitriledithiolate) and one half of 3,3-dimethyl-1,1-(propane-1,3-diyl)diimidazol-1-ium dication in the asymmetric unit. The Ni center in the [Ni(*mnt*)_2_]^2-^ anion is coordinated by four S atoms of two *mnt*^2-^ ligands, and exhibits the expected square-planar coordination geometry. The bond lengths and angles in the anion are in good agreement with those observed in other [Ni(*mnt*)_2_]^2-^ complexes (Ni *et al.*, 2004; Ren *et al.*, 2004, 2008; Duan *et al.*, 2010).

### Experimental

All reagents and chemicals were purchased from commercial sources and used without further purification. The starting materials disodium maleonitriledithiolate and 1-methyl-3-(3-(1-methyl-imidazole-3-yl)propyl)-imidazolinium iodide were synthesized following the literature procedures (Davison & Holm, 1967; Yao *et al.*, 2008). Disodium maleonitriledithiolate (456 mg, 2.5 mmol) and nickel chloride hexahydrate (297 mg, 1.25 mmol) were mixed under stirring in water (20 ml) at room temperature. Subsequently, a solution of 1-methyl-3-(3-(1-methyl-imidazole-3-yl)propyl)-imidazolinium iodide (1.5 mmol) in methanol (10 ml) was added to the mixture, and the red precipitate that immediately formed was filtered off, and washed with methanol. The crude product was recrystallized in acetone (20 ml) to give red block crystals.

### Refinement

The C-bound H atoms were placed in geometrically idealized positions with C—H bond lengths fixed to 0.97 (methylene CH_2_) 0.96 (methyl CH_3_), and 0.93 Å (aromatic CH), and refined as riding atoms. Isotropic displacement parameters of the H atom were set at *U*_iso_(H) = 1.2*U*_eq_(C) for methylene and aromatic H atoms, and *U*_iso_(H) = 1.5*U*_eq_(C) for methyl H atoms.

### Crystal data

**Table:**

(C_11_H_18_N_4_)[Ni(C_4_N_2_S_2_)_2_]	*Z* = 4
*M**_r_* = 545.38	*F*(000) = 1120.0
Monoclinic, *C*2/*c*	*D*_x_ = 1.508 Mg m^−^^3^
Hall symbol: -C 2yc	Mo *K*α radiation, λ = 0.71073 Å
*a* = 19.3683 (15) Å	µ = 1.18 mm^−^^1^
*b* = 7.3026 (6) Å	*T* = 293 K
*c* = 17.5170 (14) Å	Block, red
β = 104.167 (1)°	0.4 × 0.3 × 0.3 mm
*V* = 2402.2 (3) Å^3^	

### Data collection

**Table:**

Bruker SMART CCD area-detector diffractometer	2938 independent reflections
Radiation source: fine-focus sealed tube	2503 reflections with *I* > 2σ(*I*)
graphite	*R*_int_ = 0.067
φ and ω scans	θ_max_ = 28.3°, θ_min_ = 2.2°
Absorption correction: multi-scan (*SADABS*; Bruker, 1999)	*h* = −19→25
*T*_min_ = 0.702, *T*_max_ = 0.741	*k* = −8→9
7551 measured reflections	*l* = −23→16

### Refinement

**Table:**

Refinement on *F*^2^	Primary atom site location: structure-invariant direct methods
Least-squares matrix: full	Secondary atom site location: difference Fourier map
*R*[*F*^2^ > 2σ(*F*^2^)] = 0.037	Hydrogen site location: inferred from neighbouring sites
*wR*(*F*^2^) = 0.101	H-atom parameters constrained
*S* = 1.06	*w* = 1/[σ^2^(*F*_o_^2^) + (0.0544*P*)^2^ + 0.2056*P*] where *P* = (*F*_o_^2^ + 2*F*_c_^2^)/3
2938 reflections	(Δ/σ)_max_ < 0.001
147 parameters	Δρ_max_ = 0.31 e Å^−^^3^
0 restraints	Δρ_min_ = −0.68 e Å^−^^3^

### Fractional atomic coordinates and isotropic or equivalent isotropic displacement parameters (Å^2^)

**Table:**

	*x*	*y*	*z*	*U*_iso_*/*U*_eq_	Occ. (<1)
Ni1	0.2500	0.7500	0.0000	0.03604 (12)	
S1	0.34431 (2)	0.65936 (7)	0.08669 (3)	0.04682 (15)	
S2	0.18479 (2)	0.72779 (7)	0.08353 (3)	0.04539 (14)	
N1	0.40853 (10)	0.5664 (3)	0.29918 (11)	0.0762 (6)	
N2	0.19161 (11)	0.6190 (3)	0.28956 (11)	0.0698 (5)	
N3	0.61640 (10)	0.7743 (2)	1.00222 (10)	0.0491 (4)	
N4	0.54547 (9)	0.8708 (2)	0.89552 (10)	0.0576 (4)	
C1	0.36589 (10)	0.5949 (3)	0.24289 (11)	0.0516 (4)	
C2	0.31437 (9)	0.6359 (2)	0.17168 (10)	0.0416 (4)	
C3	0.24477 (9)	0.6629 (2)	0.17021 (10)	0.0401 (4)	
C4	0.21663 (10)	0.6392 (3)	0.23745 (11)	0.0492 (4)	
C5	0.61229 (10)	0.8655 (3)	0.93644 (11)	0.0475 (4)	
H5A	0.6506	0.9180	0.9212	0.057*	
C6	0.68163 (15)	0.7392 (3)	1.06347 (14)	0.0661 (7)	
H6A	0.7213	0.7962	1.0491	0.099*	
H6B	0.6896	0.6096	1.0689	0.099*	
H6C	0.6767	0.7889	1.1126	0.099*	
C7	0.55032 (15)	0.7161 (4)	1.00398 (16)	0.0803 (8)	
H7A	0.5380	0.6474	1.0434	0.096*	
C8	0.50654 (15)	0.7773 (5)	0.9379 (2)	0.0919 (10)	
H8A	0.4575	0.7592	0.9231	0.110*	
C9	0.52021 (13)	0.9711 (3)	0.82143 (13)	0.0750 (7)	
H9A	0.5573	1.0547	0.8150	0.090*	
H9B	0.4792	1.0439	0.8248	0.090*	
C10	0.5000	0.8508 (4)	0.7500	0.0559 (7)	
H10A	0.4602	0.7731	0.7532	0.067*	0.50
H10B	0.5398	0.7731	0.7468	0.067*	0.50

### Atomic displacement parameters (Å^2^)

**Table:**

	*U*^11^	*U*^22^	*U*^33^	*U*^12^	*U*^13^	*U*^23^
Ni1	0.03490 (18)	0.03872 (19)	0.03238 (19)	−0.00051 (11)	0.00419 (13)	−0.00245 (11)
S1	0.0380 (2)	0.0625 (3)	0.0377 (2)	0.00491 (19)	0.00505 (18)	0.0005 (2)
S2	0.0379 (2)	0.0577 (3)	0.0397 (3)	0.00376 (18)	0.00789 (19)	0.00350 (19)
N1	0.0665 (12)	0.1072 (17)	0.0468 (10)	0.0188 (12)	−0.0018 (9)	0.0070 (11)
N2	0.0691 (11)	0.0954 (14)	0.0488 (10)	0.0063 (11)	0.0220 (9)	0.0090 (10)
N3	0.0537 (9)	0.0483 (9)	0.0413 (9)	−0.0086 (7)	0.0039 (7)	−0.0020 (7)
N4	0.0479 (9)	0.0663 (10)	0.0497 (9)	−0.0038 (8)	−0.0049 (7)	−0.0074 (8)
C1	0.0493 (10)	0.0627 (11)	0.0406 (9)	0.0054 (9)	0.0065 (8)	−0.0019 (9)
C2	0.0462 (9)	0.0413 (8)	0.0344 (8)	0.0003 (7)	0.0042 (7)	−0.0017 (7)
C3	0.0459 (9)	0.0382 (8)	0.0346 (8)	−0.0023 (7)	0.0069 (7)	−0.0022 (7)
C4	0.0503 (10)	0.0539 (10)	0.0423 (10)	0.0019 (8)	0.0091 (8)	0.0012 (8)
C5	0.0454 (9)	0.0498 (10)	0.0423 (10)	−0.0046 (8)	0.0012 (7)	0.0019 (8)
C6	0.0740 (15)	0.0568 (13)	0.0549 (14)	0.0002 (10)	−0.0086 (12)	0.0085 (9)
C7	0.0686 (16)	0.112 (2)	0.0614 (15)	−0.0278 (15)	0.0177 (13)	0.0053 (14)
C8	0.0446 (12)	0.140 (3)	0.087 (2)	−0.0210 (14)	0.0079 (13)	0.0053 (19)
C9	0.0783 (15)	0.0643 (13)	0.0611 (13)	0.0102 (12)	−0.0238 (12)	−0.0046 (11)
C10	0.0481 (14)	0.0592 (17)	0.0525 (16)	0.000	−0.0027 (12)	0.000

### Geometric parameters (Å, °)

**Table:**

Ni1—S2^i^	2.1603 (5)	C2—C3	1.356 (3)
Ni1—S2	2.1603 (5)	C3—C4	1.424 (3)
Ni1—S1	2.1732 (4)	C5—H5A	0.9300
Ni1—S1^i^	2.1732 (4)	C6—H6A	0.9600
S1—C2	1.7334 (18)	C6—H6B	0.9600
S2—C3	1.7369 (17)	C6—H6C	0.9600
N1—C1	1.140 (2)	C7—C8	1.334 (4)
N2—C4	1.143 (3)	C7—H7A	0.9300
N3—C5	1.316 (2)	C8—H8A	0.9300
N3—C7	1.356 (3)	C9—C10	1.500 (3)
N3—C6	1.466 (3)	C9—H9A	0.9700
N4—C5	1.318 (2)	C9—H9B	0.9700
N4—C8	1.364 (4)	C10—C9^ii^	1.500 (3)
N4—C9	1.466 (3)	C10—H10A	0.9700
C1—C2	1.426 (2)	C10—H10B	0.9700
			
S2^i^—Ni1—S2	180.00 (3)	N3—C6—H6A	109.5
S2^i^—Ni1—S1	88.018 (18)	N3—C6—H6B	109.5
S2—Ni1—S1	91.983 (18)	H6A—C6—H6B	109.5
S2^i^—Ni1—S1^i^	91.983 (18)	N3—C6—H6C	109.5
S2—Ni1—S1^i^	88.017 (18)	H6A—C6—H6C	109.5
S1—Ni1—S1^i^	180.000 (17)	H6B—C6—H6C	109.5
C2—S1—Ni1	103.27 (6)	C8—C7—N3	106.2 (2)
C3—S2—Ni1	103.64 (6)	C8—C7—H7A	126.9
C5—N3—C7	108.85 (19)	N3—C7—H7A	126.9
C5—N3—C6	125.85 (19)	C7—C8—N4	108.8 (2)
C7—N3—C6	125.3 (2)	C7—C8—H8A	125.6
C5—N4—C8	106.8 (2)	N4—C8—H8A	125.6
C5—N4—C9	124.5 (2)	N4—C9—C10	114.08 (19)
C8—N4—C9	128.5 (2)	N4—C9—H9A	108.7
N1—C1—C2	177.7 (2)	C10—C9—H9A	108.7
C3—C2—C1	121.79 (17)	N4—C9—H9B	108.7
C3—C2—S1	120.62 (13)	C10—C9—H9B	108.7
C1—C2—S1	117.55 (14)	H9A—C9—H9B	107.6
C2—C3—C4	123.12 (16)	C9—C10—C9^ii^	108.3 (3)
C2—C3—S2	120.34 (14)	C9—C10—H10A	110.0
C4—C3—S2	116.53 (14)	C9^ii^—C10—H10A	110.0
N2—C4—C3	177.4 (2)	C9—C10—H10B	110.0
N3—C5—N4	109.39 (19)	C9^ii^—C10—H10B	110.0
N3—C5—H5A	125.3	H10A—C10—H10B	108.4
N4—C5—H5A	125.3		

Symmetry codes: (i) −*x*+1/2, −*y*+3/2, −*z*; (ii) −*x*+1, *y*, −*z*+3/2.
